# Eco-Friendly Synthesis of SnO_2_-Cu Nanocomposites and Evaluation of Their Peroxidase Mimetic Activity

**DOI:** 10.3390/nano11071798

**Published:** 2021-07-10

**Authors:** Ravi Mani Tripathi, Sang J. Chung

**Affiliations:** 1 School of Pharmacy, Sungkyunkwan University, 2066 Seoburo, Jangan-gu, Suwon, Gyeonggido 16419, Korea; rmtripathi02@gmail.com; 2 Amity Institute of Nanotechnology, Amity University Uttar Pradesh, Sector 125, Noida 201303, India

**Keywords:** green synthesis, SnO_2_-Cu nanocomposites, nanozyme, peroxidase mimetic activity, colorimetric detection, 3,3′,5,5′-tetramethylbenzidine

## Abstract

The enzyme mimetic activity of nanomaterials has been applied in colorimetric assays and point-of-care diagnostics. Several nanomaterials have been exploited for their peroxidase mimetic activity toward 3,3′,5,5′-tetramethylbenzidine (TMB) in the presence of hydrogen peroxide. However, an efficient nanomaterial for the rapid and strong oxidation of TMB remains a strategic challenge. Therefore, in this study, we developed copper-loaded tin oxide (SnO_2_-Cu) nanocomposites that rapidly oxidize TMB. These nanocomposites have strong absorption at 650 nm and can be used for highly sensitive colorimetric detection. An environmentally friendly (green), rapid, easy, and cost-effective method was developed for the synthesis of these nanocomposites, which were characterized using ultraviolet-visible, energy-dispersive X-ray, and Fourier-transform infrared spectroscopy, as well as scanning electron microscopy. This is the first green synthesis of SnO_2_-Cu nanocomposites. Their enzyme mimetic activity, which was first studied here, was found to be strongly dependent on the temperature and pH value of the solution. The synthesized nanocomposites have the advantages of low cost, high stability, and ease of preparation for enzyme mimetic applications. Hence, SnO_2_-Cu nanocomposites are a promising alternative to peroxidase enzymes in colorimetric point-of-care diagnostics.

## 1. Introduction

Nanomaterials and nanocomposites have attracted the attention of scientists owing to their unique magnetic, chemical, optical, and electrical properties, which make them suitable for various applications, such their use as catalysts [1,2], photocatalysts, drug-delivery systems [3,4,5], colorimetric sensors [6], and antibacterials [7,8]. Semiconductors and metallic nanostructures have been extensively applied in various fields. However, semiconductor–metal hybrids show improved magnetic, chemical, optical, and electrical properties compared to their independent counterparts owing to the impregnation of noble metal nanoparticles on the surface of the semiconductor–metal hybrids. Furthermore, the synthesis of nanocomposites with controllable sizes, shapes, and surface properties is important for various practical applications [9]. Zinc oxide (ZnO)-based nanocomposites are the most exploited in various fields because of their wide band gap (3.6 eV) and large exciton binding energy (130 meV) [10]. However, the combination of noble metals with tin oxide (SnO_2_) is a promising approach for enhancing the physical and chemical properties of nanocomposites. Copper (Cu) ions are suitable to be used for the catalytic activity required for the oxidation of the peroxidase substrate 3,3,5,5-tetramethylbenzidine (TMB) [11] and its nanoflowers have shown excellent activity in the catalytic detoxification of dyes [12]. Therefore, Cu-loaded (SnO_2_-Cu) nanocomposites are potential nanomaterials for chemical catalysis and other applications. Copper nanoparticles are very attractive because of their high natural abundance, low cost, and excellent catalytic, optical, electrical, mechanical, and antifungal/antibacterial properties [13,14]. Various approaches have been exploited to develop SnO_2_ and Cu nanohybrids, such as the magnetron sputtering method [15] and the chemical coprecipitation method [16]. Environment-friendly (green) synthesis methods have attracted researchers’ attention as the ideal chemical and physical synthesis methods for nanomaterials [17]. Therefore, in the present study, a green synthesis method was used for the development of SnO_2_-Cu nanocomposites. A previous study has proven that palladium-loaded ZnO (ZnO-Pd) nanosheets show peroxidase mimetic activity, oxidizing the colorless 3,3,5,5-tetramethylbenzidine (TMB) substrate in the presence of hydrogen peroxide (H_2_O_2_); the oxidized TMB (oxTMB) showed dark-blue color and a strong peak at 650 nm [18].

Horseradish peroxidase (HRP) catalyzes the oxidation of a substrate in the presence of H_2_O_2_, which acts as an electron acceptor. Due to its excellent properties, HRP is the most extensively employed enzyme in a variety of biochemical applications, such as chemiluminescence, colorimetry, and fluorimetry [19]. Enzymes have inherent drawbacks such as high preparation and purification costs, low operational stability, sensitivity to environmental conditions, and difficulties in recycling. To overcome these drawbacks, researchers are developing nanomaterials with peroxidase-like activities, functioning as artificial substitutes for enzymes with high stability. Various nanomaterials, such as gold nanoparticles [20], Pd nanoclusters [21], graphene oxide [22], and ZnO-Pd nanosheets have been developed [18]. Nanozymes (enzyme-mimicking nanoparticles) show excellent activity under harsh conditions of pH and temperature, and are resistant to protease digestion [18].

In the present study, we focused on the development of an easy, cost-effective, and green method for the biosynthesis of SnO_2_-Cu nanocomposites using a premature seed pod extract of *Platycladus orientalis*, and then investigated the nanocomposites’ enzyme mimetic activity for the oxidation of TMB. *Platycladus* species are enriched sources of carbohydrates, alkaloids, glycosides, flavonoids, tannins, and saponins [23]. These biomolecules are important sources for the biosynthesis of nanomaterials [24,25,26]. *Platycladus* species extracts have already been used for the synthesis of copper nanoparticles and reduced graphene oxide [27,28]. The synthesized SnO_2_-Cu nanocomposites were then characterized using various techniques and were found to have excellent peroxidase mimetic activity through their rapid oxidation of TMB with strong absorption at 650 nm.

## 2. Materials and Methods

### 2.1. Materials

Tin chloride dihydrate (SnCl_2_•2H_2_O) and copper sulfate pentahydrate (CuSO_4_•5H_2_O) were purchased from Sigma–Aldrich (St. Louis, MO, USA) and used as precursors for the synthesis of the SnO_2_-Cu nanocomposites. The chromogenic substrate TMB was also acquired from Sigma–Aldrich for the analysis of the enzyme mimetic activity of the SnO_2_-Cu nanocomposites. H_2_O_2_ was obtained from Samchun Chemical Co., Ltd. (Seoul, Korea).

### 2.2. Preparation of Pod Extract

Fresh premature *P. orientalis* seeds were collected from the campus of Sungkyunkwan University, Gyeonggi-do, Republic of Korea. The extract was prepared by adding 33.9 g of premature *P. orientalis* seed pods, that were thoroughly washed, dried, and chopped into fine pieces, to 100 mL of deionized water in a 250 mL Erlenmeyer flask. The mixture was boiled at 80 °C for 60 min before decanting. The solution was then cooled and filtered using Whatman paper number 1. The obtained filtrate was collected and stored at 4 °C for use in the preparation of SnO_2_-Cu nanocomposites.

### 2.3. Biosynthesis of SnO_2_-Cu Nanocomposites

Tin chloride dihydrate (0.05 M) was dissolved in 50 mL of deionized water. An aqueous solution of tin chloride dihydrate was stirred with a magnetic stirrer at 65 °C and 360 rpm. After 15 min, 5 mL of pod extract was added dropwise with continuous stirring. The solution was stirred continuously at 65 °C for 60 min. The nanostructure of SnO_2_ was separated from the aqueous medium by centrifugation at 3500 rpm for 15 min.

The purified nanostructure of SnO_2_ was re-dispersed in 50 mL of deionized water and the solution was stirred with a magnetic stirrer at 85 °C and 360 rpm. Subsequently, 5 mM copper sulfate pentahydrate was added to the above solution. The solution was stirred for 15 min at 85 °C and 360 rpm, and then 5 mL of pod extract was added to the reaction mixture. The solution was stirred continuously at 85 °C for 60 min. The pH of the reaction solution was maintained at 9 ± 1 using a 0.1 M NaOH solution.

### 2.4. Characterization of SnO_2_-Cu Nanocomposites

The biosynthesized SnO_2_-Cu nanocomposites were analyzed by ultraviolet–visible (UV–vis) spectroscopy (UH-5300, Hitachi, Ibaraki, Japan) with a scanning range of 300–800 nm. The nanocomposites were analyzed using dynamic light scattering (DLS; Zetasizer Nano S90, Malvern, UK) to determine their size distribution profile and zeta potential values. The structural characteristics of the biosynthesized SnO_2_-Cu nanocomposites were determined using scanning electron microscopy (SEM, Zeiss EVO 18, Jena, Germany). The elemental composition of the SnO_2_-Cu nanocomposites was determined by energy-dispersive X-ray spectroscopy (EDX), Pegasus2040 (EDAX, Mahwah, NJ, USA)**.** The X-ray diffraction patterns were analyzed with an X-ray Diffractometer (X’Pert PRO, PANanalytical, Netherland) with CuKα radiation (λ = 1.5417 Å) with 40 KV and 30 mA. The participation of biological molecules in the synthesis of nanocomposites was analyzed using a Fourier-transform infrared (FTIR; FTS 7000, Varian, Australia) spectroscope in the scanning range of 500–4000 nm. All characterizations were performed using standard operating procedures.

### 2.5. Evaluation of Peroxidase Mimetic Activity

The peroxidase mimetic activity of the SnO_2_-Cu nanocomposites was evaluated by the catalytic oxidation of the peroxidase chromogenic substrate TMB. The working concentration solution of H_2_O_2_ (20 mM) was prepared by diluting the purchased solution with deionized water. Dimethyl sulfoxide (DMSO) was used to prepare the TMB solution. The reaction mixture consisted of 0.525 mM TMB, 20 mM H_2_O_2_, and 0.001 mg/mL SnO_2_-Cu nanocomposites. The reaction was performed in an acetate buffer at pH 4 and incubated at 25 °C. The colorless TMB solution was converted into a dark-blue oxTMB solution, and the intensity of this color was measured by considering the absorption peak at 650 nm.

### 2.6. Effect of Buffer pH

The effect of buffer pH was determined in the range 2 to 6. Acetate buffer was used for pH 3.5, 4, and 5, whereas glycine buffer was used for pH 2, and phosphate buffer for pH 6. All buffer systems were prepared at a concentration of 0.4 M. In the reaction (total volume: 1 mL), 0.525 mM TMB (5 μL from the stock solution prepared in DMSO), 20 mM H_2_O_2_ (50 μL from stock), and 40 μL (0.001 mg/mL) of SnO_2_-Cu nanocomposites were combined, and the final volume was reached by adding acetate buffer. The reaction mixture was incubated for 10 min. The impact of buffer salt concentrations in the range of 0.1 M to 0.5 M was also evaluated.

### 2.7. Effect of Temperature

The total assay volume (1 mL) consisted of 0.525 mM TMB (5 μL from a stock solution prepared in DMSO), 20 mM H_2_O_2_ (50 μL from stock), and 40 μL (0.001 mg/mL) of SnO_2_-Cu nanocomposites, and the remaining volume was 0.1 M acetate buffer (pH 5). Temperatures from 5 °C to 80 °C were applied to determine the effect of temperature on the oxidation of TMB.

### 2.8. Effect of Assay Incubation Time

In a typical reaction (total volume of 1 mL), 0.525 mM TMB (5 μL from a stock solution prepared in DMSO), 20 mM H_2_O_2_ (50 μL from stock), and 40 μL (0.001 mg/mL) of SnO_2_-Cu nanocomposites were combined, and the final volume was maintained by adding 0.1 M acetate buffer (pH 5). The samples were scanned at 500–800 nm in the UV–vis spectrophotometer at intervals of 5 min from the incubation times of 0 min to 30 min.

## 3. Results and Discussion

### 3.1. Ultraviolet–Visible (UV–vis) Spectroscopic Analysis

**Figure 1:**
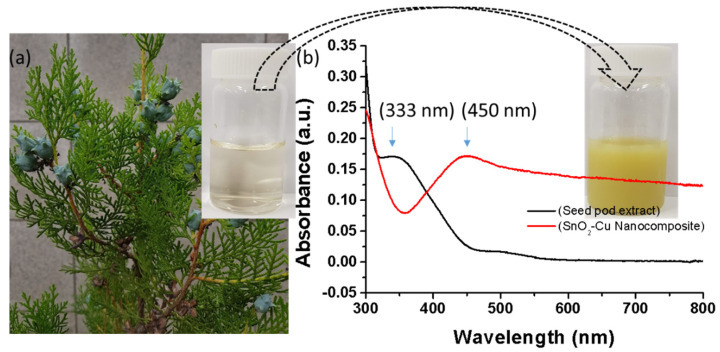
Biosynthesis of SnO_2_-Cu nanocomposites. (**a**) *Platycladus orientalis* plants with premature seed pod; the inset shows the pod extract; (**b**) ultraviolet–visible (UV–vis) spectrum of the biosynthesized nanocomposites; the inset shows a colloidal solution of the nanocomposites.

The UV–vis spectroscopy was used to determine the biosynthesis of SnO_2_-Cu nanocomposites. The SnCl_2_•2H_2_O was taken to synthesized SnO_2_ nanoparticles after interaction with pod extract at 65 °C and 360 rpm. After the interaction with leaf extract the solution color was changed and nanoparticles were precipitated. The precipitated nanoparticles were re-dispersed into 50 mL deionized water and 5 mM copper sulfate pentahydrate was added. After the addition of 5 mL of pod extract at 85 °C, maintaining pH at 9 ± 1, the color of the solution changed (Figure 1). Figure 1a shows that the *P. orientalis* plants having premature seeds pod and inset showing pod extract. Figure 1b inset shows colloidal solution of biosynthesized SnO_2_-Cu nanocomposites. The UV–vis absorption spectrum of leaf extract showed a peak at 333 nm (Figure 1b). The peak at 450 nm indicated the presence of SnO_2_-Cu nanocomposites.

The optical band gap (*E_g_*) values determined were using the Tauc method [29,30]. The direct *E_g_* for bulk SnO2 occurs at 3.60 eV [29].

(1) $$\alpha h\nu =A{\left(h\nu -E_{g}\right)}^{\gamma }$$

where *A* is a material-dependent constant, h is Planck’s constant, and *ν* is the light frequency. The power coefficient γ is characteristic of the type of transition considered, with a value of 1/2 or 2 depending on whether the transition is directly or indirectly allowed.

α is the absorption coefficient and it is calculated by the equation given below;

(2) $$\alpha =\frac{4\pi k}{\lambda }$$

Here, *k* is the extinction coefficient [30].

**Figure 2:**
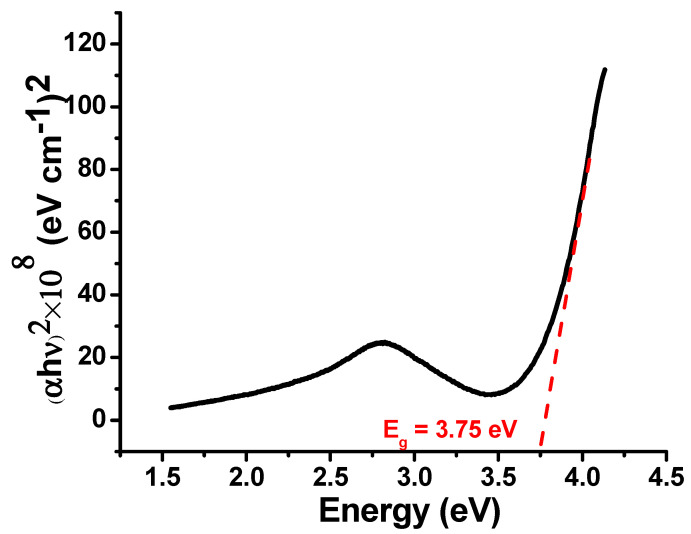
Band gap determination of biosynthesized SnO_2_-Cu nanocomposite.

From the plot (Figure 2) it is found that the synthesized SnO_2_-Cu nanocomposites have a band gap of 3.75 eV [29,31].

### 3.2. Dynamic Light Scattering (DLS) Analysis

The zeta potential and size distribution profile of the biosynthesis of the SnO_2_-Cu nanocomposites were analyzed by DLS. The as-synthesized nanocomposites had an average diameter of 738.9 nm with a polydispersity index of 0.221 (Figure S1a). The surface charge on the nanocomposites determined by DLS was −30.5 mV zeta potential, which clearly showed that the nanocomposites had a negative charge (Figure S1b). The zeta potential was obtained at 11.1 mV z-deviation and 2.26 mS/cm conductivity. The negative potential revealed the presence of biological moieties on the surface of nanocomposites [24].

### 3.3. Field-Emission Scanning Electron Microscopy (FESEM) Analysis

**Figure 3:**
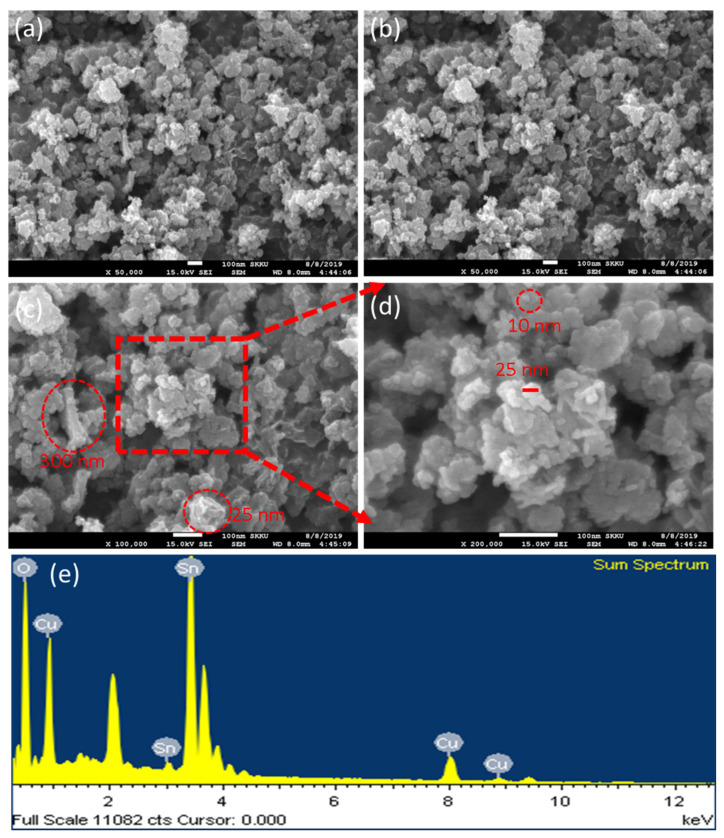
Field emission scanning electron microscope micrographs of the biosynthesized SnO_2_-Cu nanocomposites. (**a**,**b**) Overall view of samples. (**c**) Micrograph of nanocomposites. (**d**) Micrograph at high magnification. (**e**) Energy-dispersive X-ray spectrum of SnO_2_-Cu nanocomposites.

The biosynthesized SnO_2_-Cu nanocomposites were analyzed by field-emission scanning electron microscopy (FESEM) to determine their morphology and size. SnO_2_-Cu nanocomposites were freeze-dried, and the powder obtained was used to prepare samples for FESEM. The synthesized nanocomposites showed irregular structures at 50,000× magnification (Figure 3a,b). A mixture of shapes was found: rods of 25 nm (red circle in Figure 3c), sheets of 300 nm (red circle in Figure 3c), and spheres of 10 nm (red circle in Figure 3d). The synthesized nanocomposites were also scanned at 100,000× magnification, which revealed a large number of small particles with a variety of sizes, but all in the nano-dimension (Figure 3c). The nanocomposites were further scanned at high magnification (200,000×), showing some bunches less than 25 nm in size (Figure 3d).

### 3.4. Energy-Dispersive X-ray Spectroscopy (EDX) Analysis

The biosynthesized SnO_2_-Cu nanocomposites were analyzed by EDX to determine their elemental composition and purity. The EDX device was attached to the SEM instrument, which was used to obtain the EDX spectrum. The elemental profile of the biosynthesized SnO_2_-Cu nanocomposites showed strong signals for Sn, O and Cu. The spectrum did not show any other elemental signal, except for Cu, due to the Cu grid (Figure 3e). Hence, the synthesized SnO_2_-Cu nanocomposites contained pure elemental Sn, O, and Cu.

**Figure 4:**
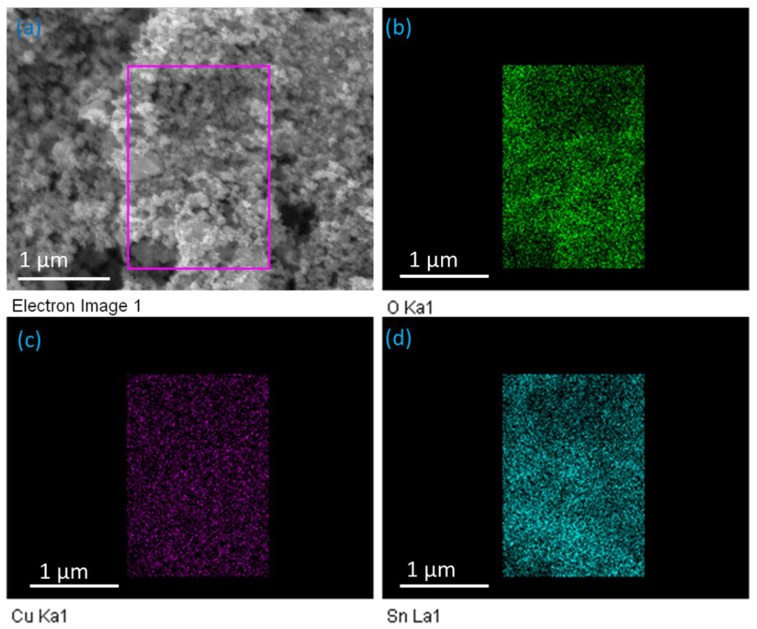
Energy-dispersive X-ray mapping of the synthesized SnO_2_-Cu nanocomposites. (**a**) Area selected in the scanning electron microscopy (SEM) image for the elemental mapping; (**b**) O-Ka1 map (green); (**c**) Cu-Ka1 map (magenta); (**d**) Sn–La1 map (blue).

Further, EDX mapping was performed to determine the distribution of Sn, O, and Cu in the nanocomposites (Figure 4a–d). An area was selected in the SEM micrograph for the EDX mapping (Figure 4a). A uniform distribution of Cu was observed in the nanocomposite (Figure 3c). The distribution of Sn and O was broader than that of Cu (Figure 4b,d), which clearly showed that Cu was densely captured in the SnO_2_ lattice. No other elements existed in the nanocomposites, confirming that no other impurities existed in the sheets. Hence, the EDX pointer and mapping confirmed the hybrid nature of the SnO_2_-Cu nanocomposites.

### 3.5. X-ray Diffraction (XRD) Analysis

**Figure 5:**
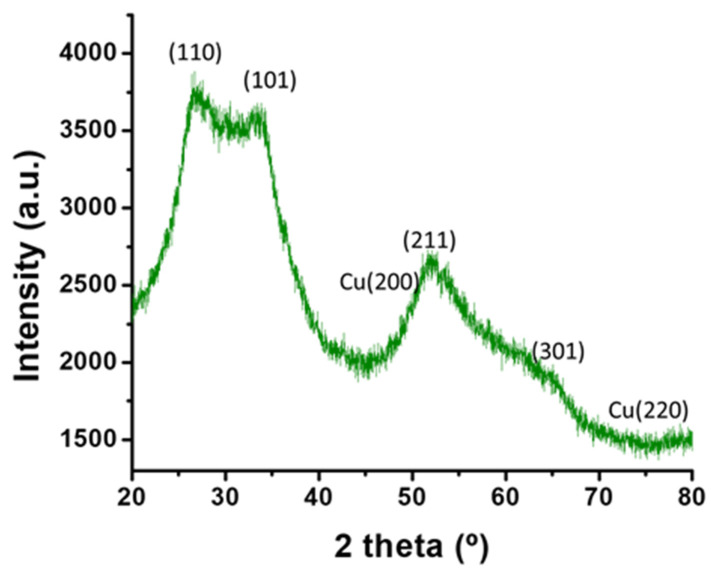
X-ray diffraction pattern of SnO_2_-Cu nanocomposites.

The powder sample of SnO_2_-Cu nanocomposites was developed by freeze drying the colloidal solution. The XRD pattern of SnO_2_-Cu nanocomposites shows diffraction angle 26.7°, 33.9°, 51.8° and 66.1°, which corresponded to (110), (101), (211), and (301), respectively (Figure 5). These reflections are characteristic of cassiterite crystal phase with tetragonal rutile structure (Joint Committee on Powder Diffraction Standards data card No. 41-1445). The highest intense peak observed at diffraction angle 26.7° (110), which reveals the preferred direction for the growth of nanocrystals. The broadness in the XRD pattern clearly indicates the presence of secondary metal in the synthesized nanostructure [32]. The diffraction peaks at 50.5° and 74.1°, which corresponded to the (200) and (220) planes of fcc structure of pure Cu (Joint Committee on Powder Diffraction Standards data card No. 71-4610). The SnO_2_ and copper have peaks at 51.8° and 50.5° which merge and create broadness. The earlier investigates have been found peaks in XRD for both phases in nanocomposites [33,34]. We also observed peaks for SnO_2_ and copper in the nanocomposite. Hence, the XRD diffraction peaks confirm the synthesized nanocomposites are hybrids of SnO_2_ and copper.

### 3.6. Fourier Transform Infrared Spectroscopy (FTIR) Analysis

Fourier transform infrared spectroscopy was performed to analyze the participation of biological molecules in the stabilization of the nanocomposites. The biosynthesized SnO_2_-Cu nanocomposites were scanned from 650 to 4000 cm^−1^ (Figure S2). The FTIR spectrum showed a strong and broad peak at 3330 cm^−1^, corresponding to the –OH stretching vibrations of the OH units and water [24]. A strong peak was observed at 1640 cm^−1^, indicating the bond for (N–H) bending, which corresponds to primary amines [18]. Another peak was observed at 665 cm^−1^, corresponding to the Sn–O stretching vibrations. Therefore, the FTIR spectrum confirmed that the biological molecules present in the pod extract contributed to the synthesis of the nanocomposites.

### 3.7. Enzyme Mimetic Activity

The peroxidase chromogenic substrate TMB has been used in various clinical diagnostic laboratories. TMB is a colorless substrate, but in the presence of H_2_O_2_, the peroxidase enzyme oxidises it in the blue diamine form. Therefore, peroxidase enzymes are extensively used for detection purposes, but they do not work in harsh pH and temperature conditions. Moreover, production and purification are time-consuming and costly. Hence, SnO_2_-Cu nanocomposites have been developed to evaluate peroxidase mimetic activity. An acetate buffer with a pH of 4 was used to perform the reaction, and after 20 min of incubation at room temperature, the solution turned blue in the presence of H_2_O_2_ and the SnO_2_-Cu nanocomposites. Figure S3 shows the UV–vis spectrum of the blue solution; the strong peak at 650 nm clearly demonstrates the characteristics of the oxidized TMB.

The colorimetric method for the detection of H_2_O_2_ using biologically synthesized SnO_2_-Cu nanocomposites proposed here is based on the premise that the peroxidase mimetic activity of SnO_2_-Cu nanocomposites originates from the abundance of Cu, which enables electron transfer through the disintegration of H_2_O_2_ to form •OH radicals and catalyzes the oxidation of TMB.

### 3.8. Effects of Buffer pH and Concentration

**Figure 6:**
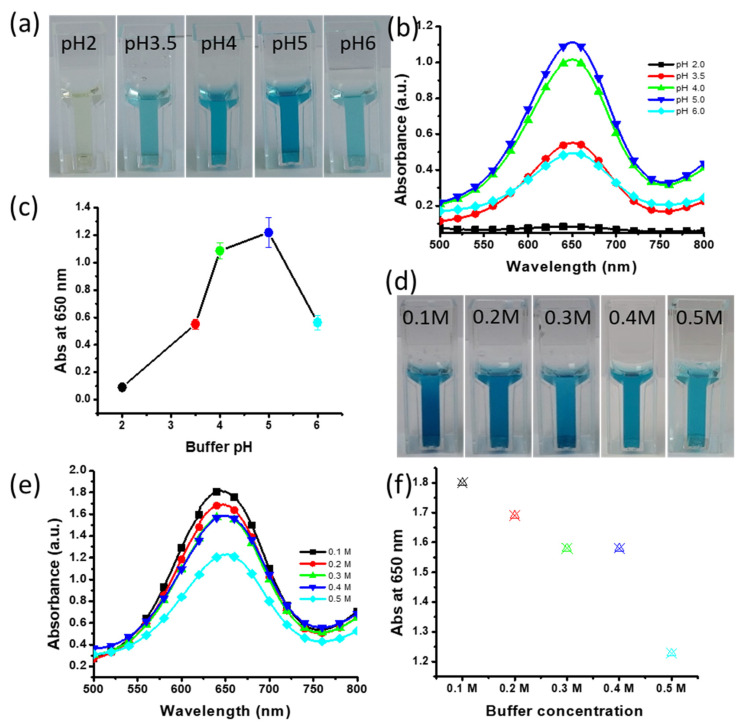
Effects of buffer pH and salt concentration. (**a**) The effect of pH on the assay color; (**b**) absorbance spectra at various buffer pH; (**c**) triplicate experiments were performed for determining the effect of buffer pH; error bars represent standard deviations; (**d**) the effect of buffer salt concentration on the assay color; (**e**) absorbance spectra at various buffer salt concentrations; (**f**) absorbance at 650 nm showing the effect of buffer concentrations.

pH plays an important role in the sensitivity of the detection assay because chromogenic detection methods work most efficiently at a specific pH. Thus, the assay was performed at pH 2–6 to determine the level of color development at each tested pH. High color intensity was observed at pH 5 (Figure 6a). Sufficient color was obtained at pH 3.5, 4, 5 and 6; however, no blue color was present at pH 2 (Figure 6a). This indicated that all pH levels, except for pH 2, are favorable for the oxidation of TMB. The highest absorption was found at pH 5 (Figure 6b,c). We further determined the impact of the buffer salt concentration on the oxidation of TMB and the development of color. As pH 5 was found to be the optimal pH for the development of the strongest color, we prepared a buffer of pH 5 with different concentrations of salt ranging from 0.1 to 0.5 M. The strongest color intensity was found for the buffer prepared with 0.1 M salt (Figure 6d). The resulting UV–vis spectra indicated that the buffer prepared with 0.1 M salt was suitable for the oxidation of TMB (Figure 6e,f). Therefore, a buffer with pH 5 and a salt concentration of 0.1 M is optimal for obtaining the strongest color intensity.

### 3.9. Effect of Incubation Temperature

**Figure 7:**
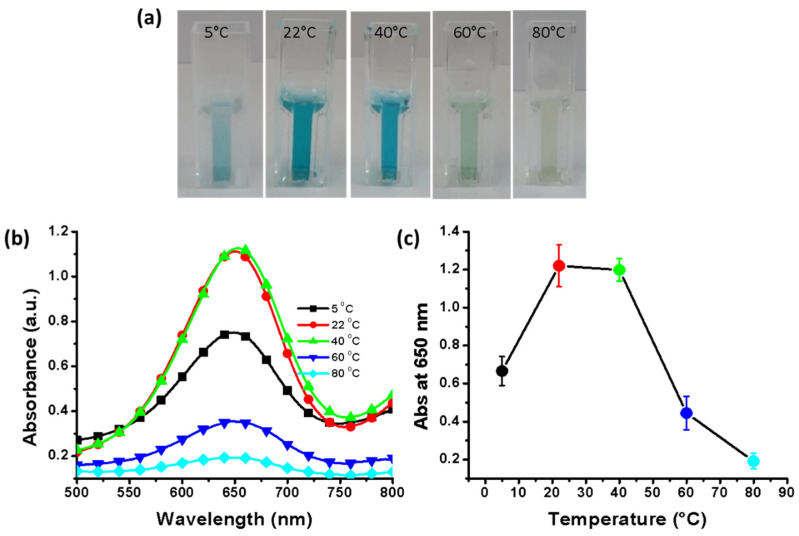
Effects of incubation temperature. (**a**) effect of temperature on color intensity; (**b**) absorbance spectra at various temperatures; (**c**) triplicate experiments were performed to determine the effect of temperature; error bars represent standard deviations.

Previous studies have reported that temperature plays a key role in the oxidation of TMB. Molybdenum disulfide and copper sulfide nanostructures have been used to oxidize TMB at optimal temperatures of 50 °C and 45 °C, respectively [35,36]. This indicates that each catalyst works most effectively at a specific temperature. Therefore, we determined the optimal temperature for the synthesized SnO_2_-Cu nanocomposites. Temperatures from 5 °C to 80 °C were evaluated, and it was observed that temperatures of 22 °C to 40 °C resulted in a strong color intensity (Figure 7a,b). The UV–vis spectra showed that the absorbance increased with increasing temperature; however, above 40 °C, the absorbance decreased drastically, and the lowest absorbance was observed at 80 °C (Figure 7b). Strong absorbance signals were observed at 22 °C and 40 °C and the highest absorbance value was obtained at 22 °C (Figure 7c). Hence, the synthesized SnO_2_-Cu nanocomposites are efficient catalysts for peroxidase mimetic activity because their highest activity was observed at room temperature, i.e., 22 °C.

### 3.10. Effect of Incubation Time

**Figure 8:**
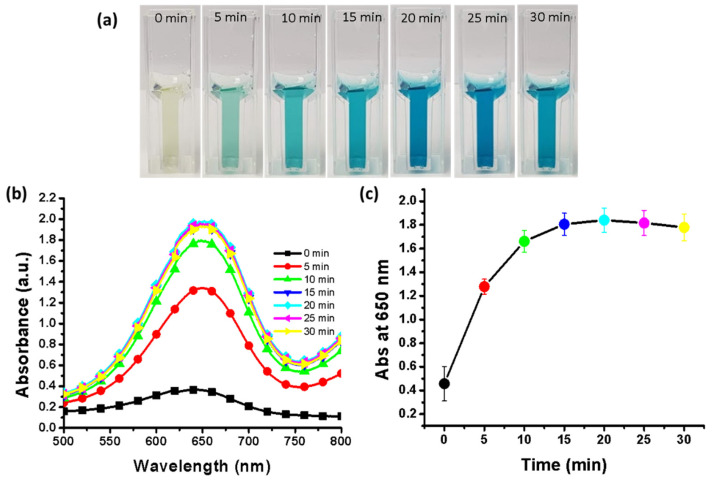
Effect of incubation time. (**a**) effect of incubation time on assay color; (**b**) absorbance spectra at various incubation times; (**c**) triplicate experiments were performed to determine the effect of incubation time; error bars represent standard deviations.

The incubation time is dependent on the type of catalyst used for the oxidation of the chromogenic peroxidase substrate TMB. In the present study, we also evaluated the optimal incubation time in the range of 0–30 min. Figure 8a shows that with increasing incubation time, the color intensity also increased; however, after 20 min, no further increase in the color intensity was observed. The samples were scanned with a UV–vis spectrophotometer in the range of 500–800 nm at intervals of 5 min for incubation times from 0 min to 30 min. The spectra showed that the absorbance did not increase after 20 min of incubation (Figure 8b). In fact, for incubation times greater than 20 min, the absorbance decreased (Figure 8c). Therefore, the experimental results support an incubation time of 20 min. Pan et al. [37] reported that the enzyme ficin and a zinc(II)-2-methylimidazole metal organic framework exhibited enhanced peroxidase activity; however, their method required an incubation time of 180 min. Therefore, the biosynthesized SnO_2_-Cu nanocomposites are efficient for the rapid oxidation of TMB.

## 4. Conclusions

In the present study, we developed a green method for the synthesis of SnO_2_-Cu nanocomposites using *P. orientalis* seed pods. To the best of our knowledge, this is the first report on the green synthesis of SnO_2_-Cu nanocomposites. Furthermore, this is the first study to evaluate the peroxidase mimetic activity of SnO_2_-Cu nanocomposites. The XRD pattern of the SnO_2_-Cu nanocomposites showed diffraction angles of 26.7°, 33.9°, 51.8° and 66.1°, corresponding to the (110), (101), (211) and (301) peaks, respectively. The diffraction peaks at 50.5° and 74.1° corresponded to the (200) and (220) planes of the fcc structure of pure Cu. The EDX spectrum of the biosynthesized SnO_2_-Cu nanocomposites showed strong signals for Sn, O and Cu. The synthesized nanocomposites showed excellent peroxidase activity at pH 5 in acetate buffer at room temperature (22 °C). Furthermore, a 20 min incubation time was found to improve the yield of oxTMB. Thus, the present study establishes that biosynthesized SnO_2_-Cu nanocomposites show excellent peroxidase activity, which can be used to develop an easy-to-use platform for clinical purposes.

## Supplementary Materials

The following are available online at https://www.mdpi.com/article/10.3390/nano11071798/s1, Figure S1: Dynamic light scattering of biosynthesized SnO_2_-Cu nanocomposites: (a) size distribution profile and (b) zeta potential of nanocomposites, Figure S2: Fourier transform infrared (FTIR) spectrum of biosynthesized SnO_2_-Cu nanocomposites, Figure S3: Ultraviolet–visible (UV–vis) spectrum of oxidized TMB after 20 min of incubation at room temperature.
