# Supplementary material for: Eco-Friendly Synthesis of SnO2-Cu Nanocomposites and Evaluation of Their Peroxidase Mimetic Activity
Source: Nanomaterials (Basel). 2021 Jul 10;11(7):1798. doi: 10.3390/nano11071798 (PMC8308257; doi:10.3390/nano11071798)
Supplement: Supplementary file 1 [file nanomaterials-11-01798-s001.zip › nanomaterials-1255037-supplementary.pdf]

nanomaterials

Eco-friendly synthesis of SnO_2-Cu nanocomposite and evaluation of their peroxidase-mimetic activity

Ravi Mani Tripathi [1,2] and Sang J. Chung [1,*]

1 School of Pharmacy, Sungkyunkwan University, 2066 Seoburo, Jangan-gu, Suwon, Gyeonggido 16419, Korea; rmtripathi02@gmail.com
2 Amity Institute of Nanotechnology, Amity University Uttar Pradesh, Sector 125, Noida 201303, India
* Correspondence: sjchung@skku.edu; Tel.: +82-31-290-7703; Fax +82-31-292-8800

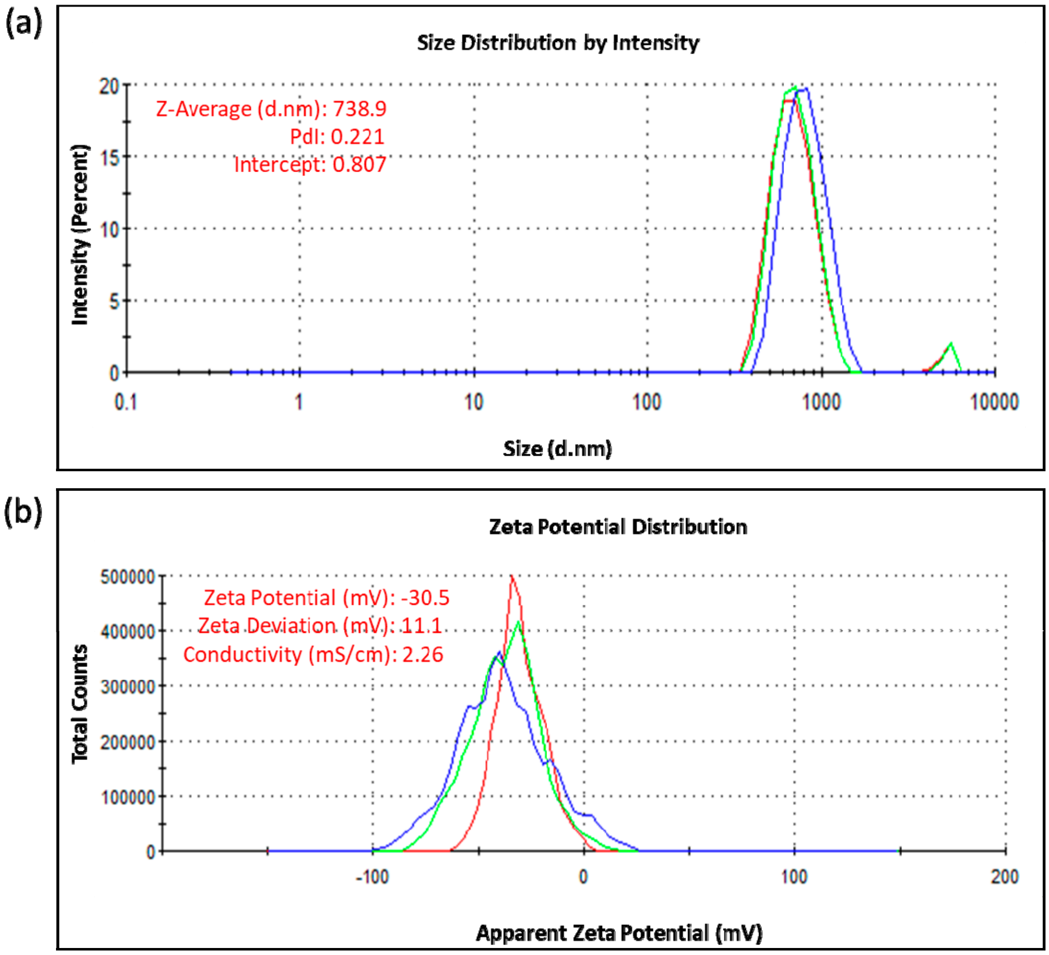

Figure S1. Dynamic light scattering of biosynthesized SnO_2-Cu nanocomposites; (a) Size distribution profile and (b) zeta potential of nanocomposites.

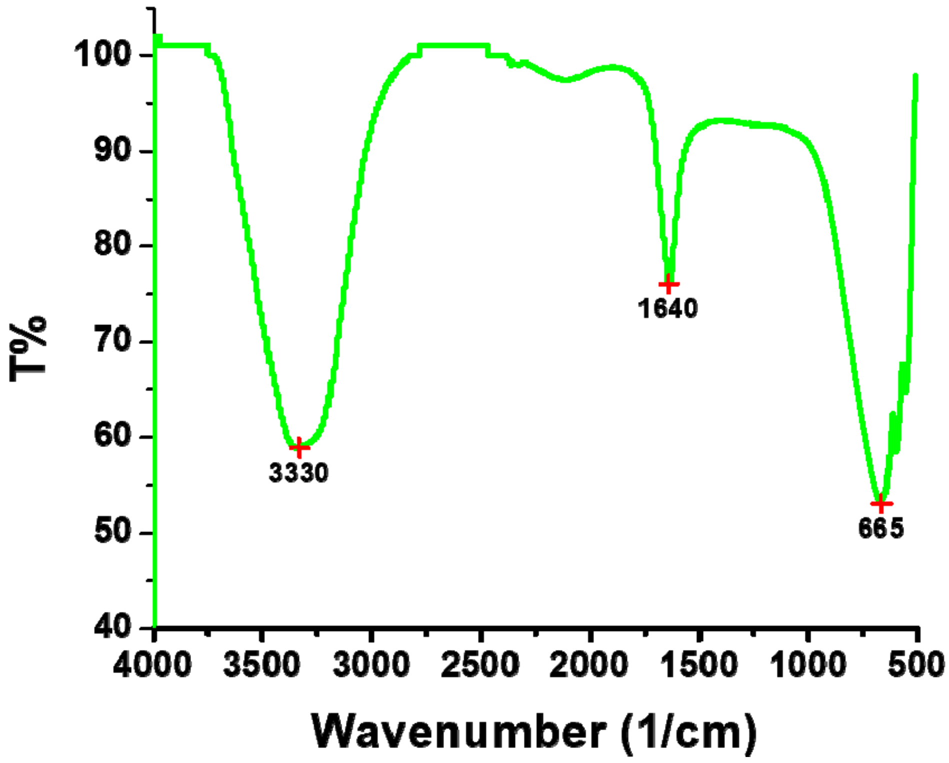

Figure S2. FTIR spectrum of biosynthesized SnO_2-Cu nanocomposites.

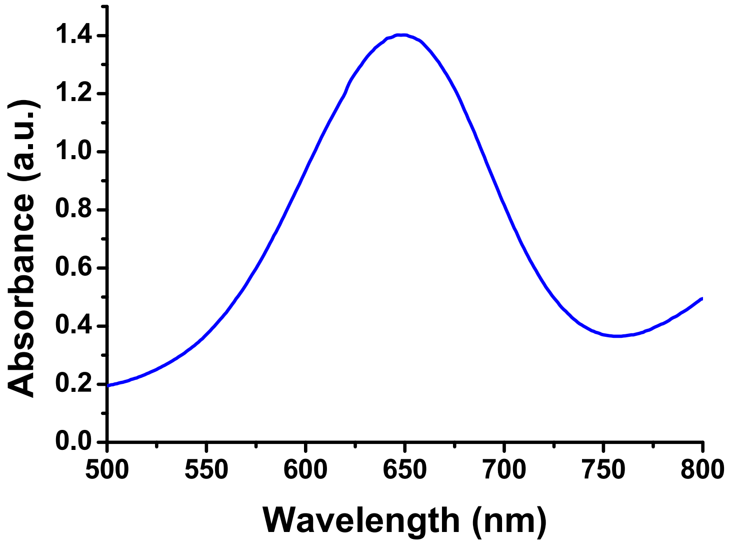

Figure S3. UV–vis spectrum of oxidized TMB after 20 min of incubation at room temperature.

Publisher's Note: MDPI stays neutral with regard to jurisdictional claims in published maps and institutional affiliations.

© 2021 by the authors. Licensee MDPI, Basel, Switzerland. This article is an open access article distributed under the terms and conditions of the Creative Commons Attribution (CC BY) license (http://creativecommons.org/licenses/by/4.0/).
